# Radionuclide Therapies in Prostate Cancer: Integrating Radium-223 in the Treatment of Patients With Metastatic Castration-Resistant Prostate Cancer

**DOI:** 10.1007/s11912-015-0495-4

**Published:** 2016-01-16

**Authors:** Sten Nilsson

**Affiliations:** Department of Oncology-Pathology, Karolinska University Hospital, Karolinska Institutet, SE-17177 Solna, Sweden

**Keywords:** Radionuclide, Alpha emitters, Beta emitters, Bone metastases, Castration-resistant prostate cancer, Radium-223 dichloride, Strontium-89, Samarium-153, Abiraterone, Enzalutamide, Docetaxel, Overall survival, Symptomatic skeletal events, Pain, Quality of life

## Abstract

Metastatic castration-resistant prostate cancer (mCRPC) frequently metastasizes to the bone, often resulting in painful skeletal events, reduced quality of life, and reduced survival. The beta-emitting radiopharmaceuticals strontium-89 and samarium-153 alleviated pain in mCRPC patients with widespread skeletal metastases and have been associated with myelotoxicity. Radium-223, a first-in-class alpha-emitting radiopharmaceutical, prolonged overall survival, delayed symptomatic skeletal events, and improved quality of life, versus placebo, in patients with CRPC and symptomatic bone metastases and no visceral metastases. Radium-223 provided survival benefit to patients with CRPC and symptomatic bone metastases, regardless of prior docetaxel use. Importantly, prostate-specific antigen level and pain palliation were not a measure of radium-223 treatment response and should not alter the decision to administer all six radium-223 injections, the recommended regimen for survival benefit. Radium-223 was generally well tolerated, leading to ongoing clinical trials in combination with other therapeutics. Thus, radium-223 is a valuable addition to the mCRPC treatment armamentarium.

## Introduction

Most patients with metastatic prostate cancer who initially respond to androgen deprivation therapy or surgical castration eventually progress to castration-resistant disease [1]. As prostate cancer transitions from castration sensitive to castration resistant, the incidence of bone metastasis increases; >90 % of patients with metastatic castration-resistant prostate cancer (mCRPC) develop bone metastases [2]. Bone metastases disrupt the homeostatic balance of bone formation and resorption, mediated by osteoblasts and osteoclasts, respectively, impairing the structural integrity of the bone and often resulting in skeletal events associated with increased pain, poor quality of life (QOL), and reduced survival [3–5].

Traditionally, the treatment strategies were aimed at managing pain and reducing skeletal complications by using analgesics, surgery, external-beam radiation therapy (EBRT), beta-emitting radionuclides, and bisphosphonates. The chemotherapeutic docetaxel was the first treatment option available to improve overall survival (OS) in patients with mCRPC [6, 7]. However, docetaxel chemotherapy is associated with severe hematologic adverse events (AEs). Ongoing research led to targeted therapeutics, such as the radiopharmaceutical radium-223 dichloride (radium-223), hormonal agents abiraterone acetate (abiraterone) and enzalutamide, and the immunotherapeutic sipuleucel-T, that improve OS in mCRPC patients [8].

Unlike systemic chemotherapy, bone-seeking radiopharmaceuticals deliver radiation directly to the bone. Available bone-seeking radionuclide therapies for patients with bone-metastatic prostate cancer are classified as beta-emitting radionuclides or alpha-emitting radionuclides (Table 1). Strontium-89 and samarium-153 are beta emitters with no proven survival benefit as single agents in mCRPC patients [9] and are recommended for pain palliation in patients with mCRPC and widespread bone metastases [10, 11]. In contrast, radium-223, a first-in-class alpha-emitting radiopharmaceutical, was shown in a large phase 3 randomized trial to prolong OS and improve time to the first symptomatic skeletal event (SSE) versus placebo [12••]. This article focuses on radium-223 and its integration into the mCRPC treatment paradigm because it is the only radiopharmaceutical that confers a survival benefit in patients with CRPC and symptomatic skeletal metastases.

**Table 1:** Physical characteristics of bone-seeking radionuclides [19, 23, 38, 50, 66]

Radiopharmaceutical	Trade name	Half-life (days)	Major emission	Linear energy transfer (MeV)	Tissue range	Indication
Beta emitters						
Strontium-89 (^89^SrCl_2_)	Metastron®	50.5	Beta	0.58	0.7 cm	Adjunct to and as an alternative to external beam radiotherapy for the palliation of pain from the bone metastases secondary to prostatic carcinoma at the stage of hormone therapy failure
Samarium-153-EDTMP (^153^Sm-EDTMP,^153^Sm lexidronam)	Quadramet®	1.9	Beta	0.22	0.33 cm	Pain relief in patients with confirmed osteoblastic metastatic bone lesions that enhance on radionuclide bone scan
Alpha-emitter						
Radium-223 (^223^RaCl_2_)	Xofigo®	11.4	Alpha	27.4	100 μm	Treatment of patients with castration-resistant prostate cancer, symptomatic bone metastases, and no known visceral metastatic disease

### Comparison of Physical Characteristics of Radionuclides: Advantages of Alpha Versus Beta Emitters

Strontium-89 and radium-223 are deposited in the bone because of their inherent calcium-mimetic nature, whereas samarium-153 lexidronam, also referred to as samarium-153-ethylenediaminetetramethylenephosphonate (EDTMP), targets the bone by chelation with EDTMP, which has a high affinity for calcium [9]. On binding to the bone, these radionuclides decay by emitting alpha or beta particles alone or with gamma rays, which kill surrounding cells. Strontium-89 is a beta emitter, and samarium-153 predominantly emits beta particles, whereas radium-223 predominantly emits alpha particles (Table 1). The goal of such irradiation is to kill tumor cells in the bone while sparing the normal bone marrow, the site of hematopoiesis; however, alpha and beta emitters are not equal in their cell-killing ability or their impact on toxicity to the bone marrow [9]. Ideally, radionuclides with a short tissue range and high linear energy transfer (LET) are likely to reduce penetration into the bone marrow and lead to targeted killing of tumor cells with minimal hematologic toxicity. Additionally, a shorter half-life is also likely to reduce adverse effects. Radium-223 meets the above criteria, and alpha particles have a relatively shorter range, spanning 2–10 cell diameters with a higher LET, thereby delivering a highly targeted effect with limited hematologic toxicity (Table 1) [13–15]. In contrast, the beta particles emitted by strontium-89 and samarium-153 have a longer range of 0.7 and 0.33 cm and lower LET of 0.58 and 0.22 MeV, respectively. The unique mechanism of action (MOA) of radium-223 underlies its efficacy. Alpha particles produce cytotoxic, predominantly nonrepairable double-stranded DNA breaks in tumor cells [13]. A recent study conducted in an osteoblastic patient-derived prostate cancer model suggests that radium-223 impacts tumor and osteoblastic bone growth [16].

### Pain Palliation With the Beta Emitters Strontium-89 and Samarium-153

Most studies with strontium-89 and samarium-153 evaluated pain palliation and were small compared with current clinical trial standards [9]. Additionally, the subjectivity of pain and differences in pain measurement make it harder to compare results across studies. A statistically significant benefit in pain palliation with strontium-89 (150 MBq) versus the stable isotope strontium-88 was observed in a small double-blind crossover trial involving 32 advanced prostate cancer patients [17]. Response was assessed 5 weeks after each treatment. In this trial, 32 patients received the first injection and 16 patients received the second injection, of whom 11 were evaluable at 5 weeks. The mean decrease in platelets from baseline after the first strontium-89 injection was 101 × 10^9^/L, and after both injections was 104 × 10^9^/L, whereas platelet count was not significantly changed in patients who received placebo as the first injection [17]. A subsequent larger, phase 3, placebo-controlled clinical trial in eight Canadian cancer centers evaluated the efficacy of a single 10.8 mCi injection of strontium-89 as an adjuvant to local-field radiotherapy in hormone-refractory mCRPC patients (*n* = 126). This trial showed that strontium-89 treatment significantly delayed pain progression. As expected from its physical characteristics, hematologic toxicity involving leukocytes and platelets was higher with the strontium-89 versus placebo group [18]. Three patients showed clinical bleed with low platelet counts (<50 × 10^9^/L); two patients (3.6 %) in the strontium-89 group versus one patient in the placebo group (2.1 %) suffered hemorrhage. Complete pain responses ranged 30–60 %. No statistically significant difference in survival was observed between treatment groups. A statistically significant reduction in prostate-specific antigen (PSA) and alkaline phosphatase (ALP) was also observed. In 1993, the US Food and Drug Administration (FDA) approved the use of strontium-89 as an adjunct and alternative to EBRT for palliation of pain from bone metastases secondary to prostate cancer at the stage of hormone therapy failure [19, 20]. The Canadian Urological Association–Canadian Urologic Oncology Group (CUA-CUOG) guidelines recommend the use of strontium-89 for pain palliation of CRPC in carefully selected patients because of the risk of prolonged myelosuppression and transfusion dependence [21].

A pivotal phase 3 trial (*n* = 152) comparing nonradioactive samarium-152 versus radioactive samarium-153 lexidronam complexes in patients with hormone-refractory prostate cancer and painful bone metastases showed that samarium-153 lexidronam 1 mCi/kg was safe and effective for pain palliation [22]. In that trial, pain intensity was measured using the linear 100-mm visual analog scale and nonlinear pain descriptor scales; a statistically significant reduction in opioid use, suggesting pain reduction, was observed at treatment weeks 3 and 4. Incidence of grade 4 hemoglobin level was 2 % in samarium-152-treated versus 1 % in samarium-153-treated patients; incidences of grade 4 platelet level and grade 4 white blood cell level were 0 % in both groups. Samarium-153 was approved for pain palliation in patients with confirmed osteoblastic metastatic bone lesions and is used in prostate cancer patients with painful bone metastases [23].

A recent systematic review and meta-analysis of clinical studies involving strontium-89 and samarium-153 showed overall efficacy of 70 % for both agents in alleviating metastatic bone pain in mCRPC patients and complete pain relief in 27 % of patients [24]. Dose-response studies showed increasing rates of response to pain and increasing myelotoxicity with increasing doses of samarium-153, limiting the use of higher doses [25]. In contrast, radium-223 showed increasing pain response with increasing doses, without obvious differences in hematologic toxicities at different doses [26].

Pain recurrence is common among mCRPC patients with skeletal metastases. Therefore, repeated administration of strontium-89 and samarium-153 has been investigated for sustained pain relief. Repeated samarium-153 administration at 1 mCi/kg in a trial involving 202 cancer patients receiving two or more doses showed reversible hematologic adverse effects and effective pain palliation; platelet and leukocyte counts returned to baseline levels by week 8 in 90 % of patients, and pain scores decreased significantly at week 4 after each of the first three doses [27]. Repeated strontium-89 administration in cancer patients with painful skeletal metastases was also safe and effective [28]. However, so far, there is no conclusive evidence supporting a survival benefit with the beta emitters strontium-89 and samarium-153 when used as single agents in mCRPC patients.

### Beta Emitters With Prior or in Combination With Chemotherapy or EBRT

To improve the effectiveness of strontium-89 and samarium-153, combinations with chemotherapeutics and EBRT have been studied. In the TRAPEZE phase 2/3 trial in 757 mCRPC patients, strontium-89 treatment after 6 cycles of docetaxel improved clinical progression-free survival (HR 0.85; 95 % CI 0.72–0.99; *p* = 0.036) [29]. A smaller phase 2 trial involving 72 mCRPC patients, who had previous induction chemotherapy and were subsequently randomized to doxorubicin alone (median survival 16.8 months) or combined with strontium-89 (median survival 27.7 months), showed a survival benefit of 10.9 months in patients treated with the combination (HR 2.76; 95 % CI 1.44–5.29) [30]. A subsequent phase 2 trial of a combination of strontium-89 with alternating chemotherapeutics and hormonal therapeutics in advanced hormone-independent prostate cancer patients also showed a benefit of strontium-89 in progression-free survival and OS [31]. A phase 2 trial of a consolidation regimen of samarium-153-EDTMP with docetaxel in mCRPC patients, who had previously responded or stabilized after docetaxel and hormonal therapy, showed that the combination was well tolerated and produced sustained pain relief and a PSA response [32]. Samarium-153 was safely used in prostate cancer patients who had prior chemotherapy or radiotherapy [33].

A recent trial of 177 mCRPC patients with multiple painful bone metastases showed that samarium-153, combined with local EBRT versus samarium-153 alone, improved pain palliation without significantly increasing hematologic and overall toxicity [34]. The concurrent use of strontium-89 with EBRT was effective and safe in a recent retrospective analysis [35]. The National Comprehensive Cancer Network (NCCN) Clinical Practice Guidelines in Oncology recommend strontium-89 or samarium-153 with or without focal EBRT for pain palliation in patients with widespread bone metastases.

### Improving Survival in Metastatic CRPC With Radium-223

Promising results from previous phase 1 and 2 studies showing a favorable safety profile and survival benefit of radium-223 treatment in mCRPC patients led to a phase 3 trial, Alpharadin in Symptomatic Prostate Cancer (ALSYMPCA) [36, 37]. This phase 3, randomized, double-blind, multinational trial compared efficacy and safety of radium-223 versus placebo in CRPC patients with symptomatic bone metastases [12••]. The trial enrolled 921 patients with histologically confirmed, progressive CRPC with two or more bone metastases and no known visceral metastases, who received docetaxel or were not healthy enough or declined docetaxel. Patients were required to have regular use of analgesic medication or treatment with EBRT for palliation of cancer-related bone pain within the previous 12 weeks. Patients were randomized 2:1 to receive six injections at 4-week intervals of either radium-223 (intravenous injection of 50 kBq per kilogram of body weight) or placebo; both groups received best standard of care (BSOC). Prior to randomization, patients were stratified by previous use of docetaxel (yes or no), baseline ALP level (<220 U/L vs. ≥220 U/L), and the current use of a bisphosphonate (yes or no) [12••]. The planned follow-up period was 3 years after each patient’s first injection [12••].

#### Efficacy: OS

The primary efficacy end point of ALSYMPCA was OS; the secondary end points included time to first SSE, change in PSA and ALP levels, and quality of life (QOL) [12••]. In the final analysis involving 921 patients, radium-223 prolonged median survival by 3.6 months (median survival, 14.9 vs. 11.3 months; HR 0.70; 95 % CI 0.58–0.83; *p* < 0.001). The radium-223 survival benefit was consistent across patient subgroups classified based on ALP levels, bisphosphonate use, opioid use, Eastern Cooperative Oncology Group performance status, prior docetaxel use or nonuse, and extent of metastases [12••]. ALSYMPCA results led to regulatory approval of radium-223 in CRPC with symptomatic skeletal metastases and no visceral metastases; the recommended treatment regimen to achieve OS benefit is six injections of radium-223 50 kBq (1.35 μCi) per kilogram of body weight, administered intravenously at 4-week intervals [10, 11, 20, 21, 38–40]. Radium-223 represents an important advancement, as the only radiopharmaceutical agent that improves OS in mCRPC patients.

#### Efficacy: SSE

In the clinical trials of therapeutics in mCRPC patients performed before ALSYMPCA, skeletal complications were described as skeletal-related events (SREs), which were monitored through periodic radiologic review. SREs included pathologic fracture and spinal cord compression (both determined by serial radiologic review), radiation therapy or surgery to the bone, and change in antineoplastic therapy to treat bone pain [41, 42]. As a secondary end point, ALSYMPCA used a more clinically relevant end point based on clinical manifestation, rather than serial radiologic review, and included time to the first *symptomatic* skeletal event (SSE), defined as use of EBRT to relieve bone pain, occurrence of a new symptomatic pathologic fracture, spinal cord compression, or tumor-related orthopedic surgical intervention. Importantly, radium-223 increased the median time to first SSE by 5.8 months compared with placebo (median 15.6 months vs. 9.8 months; HR 0.66; 95 % CI 0.52–0.83; *p* = 0.00037) [43•]. Additionally, radium-223 significantly reduced the risk of requiring EBRT for bone pain (HR 0.67; 95 % CI 0.53–0.85; *p* = 0.001) and the risk of spinal cord compression (HR 0.52; 95 % CI 0.29–0.93; *p* = 0.03) compared with placebo. Although not statistically significant, a trend toward the reduction of the risk of symptomatic pathologic bone fracture (HR 0.62; 95 % CI 0.35–1.09; *p* = 0.10) or the need for tumor-related orthopedic surgery (HR 0.72; 95 % CI 0.28–1.82; *p* = 0.48) was observed. Prespecified analyses of subgroups by baseline total ALP, previous docetaxel use, and current bisphosphonate use also showed a consistent trend favoring radium-223 treatment [43•].

#### Efficacy: Biochemical Markers

Radium-223 MOA suggests a primary pharmacodynamic effect on serum total ALP (tALP) rather than PSA, unlike hormonal agents targeting the androgen receptor (AR) axis for which PSA level can indicate therapeutic effect. Moreover, ALP has been found to be a more clinically relevant marker than PSA for bone metastasis in prostate cancer [44, 45]. Consistently, in ALSYMPCA, radium-223 treatment versus placebo led to a significantly greater proportion of patients with a decrease of at least 30 % in tALP (radium-223, 47 %; placebo, 3 %; *p* < 0.001) [12••]. Exploratory post hoc analyses of tALP in the 3-year follow-up of ALSYMPCA patients showed that radium-223 treatment led to a rapid and sustained decrease in tALP, which correlated with OS [46]. In ALSYMPCA, radium-223 treatment led to a significant increase in the median time to increase in PSA level (HR 0.64; 95 % CI 0.54–0.77; *p* < 0.001) [12••]. Although encouraging, these results of tALP and PSA dynamics in radium-223-treated patients need validation in additional clinical studies. Identification of biomarkers of radium-223 efficacy remains a pressing clinical need.

#### Efficacy: QOL and Effect on Bone Pain

Radium-223 treatment led to a clinically meaningful improvement in QOL versus placebo, as measured by Functional Assessment of Cancer Therapy—Prostate (FACT-P) score during the treatment period (25 vs. 16 %; *p* = 0.02; mean change from baseline to week 16, −2.7 vs. −6.8; *p* = 0.006) in ALSYMPCA [12••]. In a post hoc analysis, assessment of the effect of radium-223 on health-related QOL, measured by EuroQol 5D (EQ-5D) utility scores, demonstrated the greatest treatment benefit in stable disease (disease progression measured by PSA and ALP levels), prior to an SSE [47]. In conjunction with the effect of radium-223 in delaying SSEs, these results suggest the importance of earlier use of radium-223 in disease progression to prevent SSEs, which can result in decreased QOL. Post hoc analyses of ALSYMPCA FACT-P scores showed that radium-223 treatment was associated with greater odds of pain relief versus placebo at weeks 16 and 24 of treatment (odds ratio 2.58; 95 % CI 1.18–5.62; *p* = 0.018) [48]. However, pain response was not observed in all patients receiving radium-223 [48]. An ongoing phase 2 trial is assessing radium-223 efficacy in pain palliation (Table 2, NCT02278055) [49]. Thus, pain alleviation should not be used as a measure of radium-223 efficacy to guide treatment continuation. Instead, adding BSOC, such as analgesics, for pain management should be considered while continuing radium-223 treatment to ensure that patients receive all six radium-223 injections, the recommended course to achieve OS benefit [38].

**Table 2:** Currently ongoing clinical trials of radium-223 in metastatic prostate cancer [49]

Trial	Trial design	Primary and secondary end points	Trial start date, estimated trial completion date^a^, and planned enrollment
NCT02278055	A phase 2, open-label, nonrandomized trial to assess pain efficacy with radium-223 in symptomatic mCRPC within 12 weeks of treatment	Primary: pain response Secondary: changes in bone ALP and other bone biomarkers	October 2014, October, 2016, 15
NCT02023697	A three-arm, randomized, open-label phase 2 trial of radium-223 dichloride 50 vs. 80 kBq/kg, and vs. 50 kBq/kg in an extended dosing schedule in CRPC patients with bone metastasis	Primary: SSE-free survival Secondary: OS, time to first SSE, rPFS, time to radiologic progression, pain improvement rate, time to pain progression, number of patients with TEAEs or SAEs, change in 24-h analgesic use	March 2014, October 2018, 389
NCT02346526	A single-arm, open-label, phase 2 biomarker trial of radium-223 in mCRPC patients	Primary: change from baseline in bone scan index at 2 months Secondary: mean percentage change in bone lesion area by 18-month survival status, changes in CTC number, circulating biomarkers of tumor microenvironment, changes in CTC number and translational biomarkers	May 2015, July 2021, 22
NCT02204943	An interventional, open-label pharmacodynamic trial of radium-223 in CRPC patients with bone metastases	Primary: change in proportion of patients who overexpress ALP in bone metastases Secondary: change in biomarkers of epithelial plasticity and osteomimicry expressed in the metastases of men with bone metastatic CRPC	October 2014, October 2016, 20
NCT02141438, REASSURE	An observational trial in the routine clinical practice setting to evaluate the short- and long-term safety of radium-223 in mCRPC patients and to evaluate the risk of developing second primary cancers	Primary: incidence of developing second primary malignancies, incidence of treatment-emergent SAEs, incidence of drug-related TEAE, incidence of drug-related SAEs, bone marrow suppression Secondary: OS, the worst pain score and pain interference score over time as determined by patient responses on the Brief Pain Inventory (short form) questionnaire	August 2014, September 2023, 1334
NCT02034552	A randomized, open-label, phase 2a trial evaluating quantified bone scan response following treatment with radium-223 alone or in combination with abiraterone acetate or enzalutamide in CRPC patients with bone metastases	Primary: patient bone scan response Secondary: rPFS, SSE-FS, time to first SSE, OS, time to radiologic bone progression by treatment group, number of patients with TEAE, and number of patients with SAE	December 2013, June 2016, 66
NCT02463799	A phase 2, randomized of sipuleucel-T with or without radium-223 in men with asymptomatic or minimally symptomatic bone-metastatic CRPC	Primary: immune response to treatment with sipuleucel-T (with or without radium-223) measured by peripheral PA2024 T-cell proliferation Secondary: peripheral antigen-specific T-cell proliferation over time, peripheral antigen-specific T-cell activation to sipuleucel-T over time, antigen-specific antibody response to sipuleucel-T over time, sipuleucel-T-induced antigen spread (epitope spread) phenomena, the sipuleucel-T product immune parameters, safety of combined use of radium-223 and sipuleucel-T, time to PSA progression, time to ALP progression, time to pain progression and first cancer- related opioid use, time to radiographic or clinical progression, time to first SRE, time to first chemotherapy use	July 2015, December 2018, 34
NCT02043678 (ERA223)	A randomized, phase 3, double-blind, placebo-controlled trial of radium-223 dichloride in combination with abiraterone acetate and prednisone/prednisolone in the treatment of asymptomatic or mildly symptomatic chemotherapy-naïve subjects with bone predominant mCRPC	Primary: SSE-free survival Secondary: OS, time to opiate use for cancer pain, time to pain progression, time to cytotoxic chemotherapy, rPFS, number of patients with AEs	March 2014, December 2017, 800
NCT02398526, PARABO	An observational, prospective, single-arm cohort trial for pain evaluation in radium-223-treated patients with bone metastases	Primary: pain response Secondary: change of pain over time, change in bone-pain related QOL, pain control rate, pain progression rate, time to first pain progression, time to first opioid use, summary of evaluation of covariates on pain response, relation between bone uptake in known lesions and pain palliation, dosage of radium-223, number of injections of radium-223, course of blood count, number of patients with TEAE, time to next tumor treatment, time to first SSE, OS	March 2015, September 2017, 300
NCT01810770	An interventional, phase 3, single-arm, international, open-label, prospective trial to evaluate safety and efficacy of radium-223 dichloride in an Asian population with CRPC metastatic to the bone	Primary: number of patients with AEs, number of patients with laboratory changes, number of patients with changes in vital signs, number of patients with changes in ECG, OS Secondary: changes in total ALP, number of patients with total ALP normalization, time to total ALP progression, changes in PSA, time to PSA progression, time to first SRE, SRE-free survival, time to first SRE, time to occurrence of first use of radio-isotopes to relieve skeletal symptoms, time to occurrence of first start of any other anticancer treatment, time to occurrence of first deterioration of ECOG performance status, QOL	March 2013, September 2016, 234
NCT02331303	An observational trial to evaluate the extent of potential off-label use of radium-223 (Xofigo®) in Sweden	Primary: proportion of men with mCRPC of radium-223 use, proportion of being women of radium-223 use, proportion of being children of radium-223 use, proportion of bone metastasis but having a diagnosis other than mCRPC, dosage of radium-223, proportion of patients of dose outside label recommendation	April 2015, June 2016, 200
NCT02450812 (URANIS)	An observational, prospective, single-arm cohort trial to assess OS, SSE-free survival, and QOL of chemo-naïve mCRPC patients receiving radium-223 under real-life conditions in Germany	Primary: OS Secondary: evaluation of covariates on OS in mCRPC patients, SSE-FS, covariates on SSE-FS, time to next tumor treatments, incidence of TEAE, QOL, activities of daily living, body function	May 2015, November 2019, 500
NCT02194842 (mCRPC-PEACEIII) (not yet recruiting patients)	A randomized, multicenter phase 3 trial comparing enzalutamide vs. a combination of radium-223 and enzalutamide in asymptomatic or mildly symptomatic CRPC with metastasis to bone	Primary: rPFS Secondary: OS, prostate cancer-specific survival, first SSE, time and incidence of first skeletal progression-free, time from entry to initiation of next systemic therapy, treatments elected after first disease progression, second progression-free survival in sequential regimen, pain, time to pain progression, occurrence of AEs, time to first use of opioid analgesics, QOL	December 2014, October 2018, 560
NCT02396368 (not yet recruiting patients)	A phase 1/1b trial of radium-223 in combination with tasquinimod for CRPC patients with bone metastases Note: Active Biotech and Ipsen have decided to discontinue development of tasquinimod in prostate cancer (press release 4-16-15)	Primary: safety of combining radium-223 with tasquinimod Secondary: bone ALP response, time to radiographic or clinical progression or death, time to first symptomatic SRE, proportion of patients without symptomatic progression at 6 months, median change in bone scan index (BSI) within patients at 12 weeks compared to baseline bone scan, PSA progression, time to death after start of trial treatment, changes in bone markers	March 2015, July 2017, 44
NCT02456571 (not yet recruiting patients)	A pilot trial to define the relevant immune checkpoints expressed on metastatic prostate cancer circulating tumor cells (CTC)	Primary: percent expression of immune checkpoint markers on CTCs Secondary: changing prevalence of immune checkpoint biomarkers on CTCs over time in a longitudinal analysis of four different populations of men with metastatic prostate cancer, change over time in mutational profiles, androgen receptor variant expression, and immune- and tumor-related RNA signatures in CTC-enriched blood, expression of PD-L1, PD-L2, B7-H3, and CTLA-4 in metastatic tumor tissue obtained by elective CT or ultrasound-guided research biopsies in up to 10 patients and compare this expression percentage with CTC immune checkpoint expression	October 2015, January 2017, 40
NCT02225704 (ongoing trial, not recruiting patients)	A phase 2, interventional trial to determine the safety and tolerability of radium-223 administered in combination with enzalutamide in progressive mCRPC	Primary: determine safety Secondary: time to clinical and PSA progression, PSA response, time to first skeletal related event, pain assessment, OS	August 2014, December 2016, 44

Data from Clinicaltrials.gov database accessed on September 27–28, 2015

*AE*s adverse events, *ALP* alkaline phosphatase, *BSI* bone scan index, *CRPC* castration-resistant prostate cancer, *CT* computed tomography, *CTC* circulating tumor cell, *mCRPC* metastatic castration-resistant prostate cancer, *OS* overall survival, *PSA* prostate-specific antigen, *QOL* quality of life, *rPFS* radiologic progression-free survival, *SAE*s serious adverse events, *SRE* skeletal related event, *SSE* symptomatic skeletal event, *SSE*-*FS* symptomatic skeletal event-free survival, *TEAE*s treatment-emergent adverse events

^a^Estimated study completion date is the final data collection date for the primary end point

#### Safety

Of 921 patients randomized in ALSYMPCA, 901 were included in the safety population. Radium-223 safety was previously reviewed in detail for the overall ALSYMPCA population and in patient subgroups (prior docetaxel, concomitant EBRT, baseline opioid use [yes/no]) [50]. Radium-223 was well tolerated with no clinically meaningful differences in incidence of hematologic AEs between trial groups; grade 3 febrile neutropenia occurred in one patient (<1 %) in each group and grade 5 thrombocytopenia in one patient in the radium-223 group, without evidence of bleeding [12••]. Additionally, chemotherapy after radium-223 treatment appeared to be well tolerated [51, 52].

Radium-223 remained safe and well tolerated and showed no new safety concerns during the 3-year follow-up period [53]. The favorable safety profile of radium-223 observed in ALSYMPCA led to the ongoing international, prospective, observational, cohort trial to evaluate the long-term safety profile of radium-223 in a real-world setting in more diverse CRPC patients with bone metastases (REASSURE). Patients will be followed until 7 years after the last radium-223 dose, and the decision to treat with radium-223 will be independent from and before enrollment in the trial (Table 2, NCT02141438).

### Integration of Radium-223 in the Management of Patients With Metastatic CRPC

Therapeutic options for mCRPC are rapidly growing, and, in addition to radium-223, a number of therapies that prolong survival have been added to the mCRPC treatment armamentarium. The optimal use of radium-223 in managing this disease requires determining the sequencing and combination of these agents. Results of studies addressing the integration of radium-223 relative to docetaxel and hormonal agents are discussed here, with a mention of ongoing radium-223 clinical trials.

#### Sequencing of Therapeutics: Radium-223 With Concurrent EBRT

ALSYMPCA involved patients who are already receiving EBRT for bone pain at trial entry as well as those who received EBRT during the trial, as part of BSOC. A post hoc analysis evaluated the safety of concomitant EBRT with radium-223 and the effect of radium-223 on the requirement of EBRT for bone pain relief [54]. The hematologic safety profile of radium-223 with concomitant EBRT was similar to that without concomitant EBRT, providing the additional option of EBRT for pain during radium-223 treatment.

#### Radium-223 in Patients With Prior Docetaxel

At the initiation of the ALSYMPCA trial, docetaxel was the only available drug that improved survival of mCRPC patients; ALSYMPCA included patients both with and without prior docetaxel therapy. A prespecified subgroup analysis investigated radium-223 efficacy and safety by prior docetaxel use [55•]. Of the 921 randomized patients, 526 (57 %) had prior docetaxel (radium-223, 352; placebo, 174) and 395 (43 %) had no prior docetaxel (radium-223, 262; placebo, 133). Radium-223 prolonged median survival versus placebo, regardless of prior docetaxel use (prior docetaxel use, HR 0.70; 95 % CI 0.56–0.88; *p* = 0.002) or nonuse (no prior docetaxel use, HR 0.69; 0.52–0.92; *p* = 0.01) [55•]. Consistent with the known hematologic toxicity of docetaxel, patients who received prior docetaxel had a higher incidence of thrombocytopenia with radium-223 than with placebo (9 vs. 3 %), whereas among patients without prior docetaxel, the incidence of thrombocytopenia was similar between groups (radium, 3 %; placebo, 1 %). Overall, radium-223 improved OS and had a favorable safety profile with a low incidence of myelosuppression irrespective of previous docetaxel use, leading to clinical practice guideline recommendations for the use of radium-223 in mCRPC regardless of prior docetaxel use [10, 39]. Thus, radium-223 is a new treatment option for mCRPC patients who previously received docetaxel as well as those who are not healthy enough to tolerate docetaxel therapy.

#### Chemotherapy Post Radium-223

A post hoc analysis evaluated safety and OS in a subset of ALSYMPCA patients (radium-223, 142; placebo, 64) who received chemotherapy following treatment with radium-223 or placebo. There were no detrimental effects on OS and no new hematologic safety concerns, suggesting that chemotherapy may be given safely following treatment with radium-223 [56].

#### Radium-223 With Concurrent Docetaxel

Preliminary findings from a phase 1/2a trial (*N* = 46) comparing radium-223 combined with docetaxel versus docetaxel alone in mCRPC patients showed that the combination was well tolerated [57]. The trial regimen was 5 intravenous injections of radium-223 (50 kBq/kg), 1 injection every 6 weeks, and 10 intravenous infusions of docetaxel (60 mg/m^2^), once every 3 weeks [57]. Phase 1 results concluded that it was safe to proceed to the expanded phase 2a safety cohort. Preliminary phase 2a results showed that the combination of radium-223 and docetaxel remained safe and well tolerated, and levels of bone ALP and PSA decreased in both treatment arms. Intriguingly, a higher percentage of patients receiving the radium-223 and docetaxel regimen versus docetaxel alone had normalized bone ALP levels [57]. Larger studies are needed to discern the clinical benefit of such changes in serum markers.

#### Radium-223 With Concurrent Hormonal Therapies

CRPC is now known to be driven by AR signaling [58, 59]. Two agents targeting the androgen axis have been investigated in mCRPC: abiraterone and enzalutamide. In a pivotal, randomized, placebo-controlled phase 3 trial (COU-AA-301), abiraterone with prednisone significantly improved survival (14.8 vs. 10.9 months; *p* < 0.001) in mCRPC patients who had previously received docetaxel. Abiraterone was approved by the FDA in the post-docetaxel setting in April 2011 [10, 20, 60]. A phase 3 trial (COU-AA-302) of abiraterone in the pre-docetaxel setting resulted in its FDA approval in chemotherapy-naïve mCRPC patients [10, 20, 61]. Enzalutamide improved OS (18.4 vs. 13.6 months; *p* < 0.001) of mCRPC patients who had previously received docetaxel in a randomized, placebo-controlled phase 3 trial (AFFIRM), leading to its FDA approval in 2012 for treating mCRPC in docetaxel-treated patients [62]. Enzalutamide reduced the risk of death by 29 % versus placebo in chemotherapy-naïve patients (HR 0.71; 95 % CI 0.60–0.84; *p* < 0.001), leading to extension of its FDA approval to these patients [63]. These agents’ availability and their complementary MOAs suggested a potential added clinical benefit by combining them with radium-223. Importantly, abiraterone, enzalutamide, and radium-223 have non-overlapping toxicities, further reducing the possibility of serious treatment-emergent adverse events (TEAEs) resulting from the combination.

Prior to the May 2013 FDA approval of radium-223, a phase 2, prospective, interventional, open-label, multicenter US expanded access program (EAP) trial was conducted in the USA, providing early access of radium-223 to mCRPC patients with symptomatic metastases and assessing acute and long-term safety of radium-223 [64]. One hundred eighty-four patients with symptomatic, progressive, bone-predominant mCRPC and no visceral metastases, who either received or were not eligible for docetaxel, received abiraterone and enzalutamide prior to the start of, during, and following the radium-223 treatment period. Primary end points included safety in the acute period (up to 30 days posttreatment) and long-term treatment period (≥30 days posttreatment through follow-up); other and exploratory end points were OS, time to first SSE, and changes in ALP and PSA levels. This trial was terminated early following radium-223 regulatory approval, resulting in a limited follow-up period of approximately 3 to 9 months [64].

Patient demographics and baseline characteristics were generally similar in the ALSYMPCA and US EAP trials, except for total ALP and PSA levels and median time from diagnosis, which were longer in EAP patients [64]. The incidences of grades 3 to 5 TEAEs were similar in the ALSYMPCA and US EAP trials, as were the incidences of hematologic AEs. No secondary malignancies in either trial were attributed to radium-223 [64]. Patient censoring and the short follow-up period did not allow estimation of OS of all patient subgroups; patients treated concurrently with radium-223 and abiraterone/enzalutamide (*n* = 17) (without prior treatment with abiraterone/enzalutamide) appeared to survive longer than those who had not received concurrent hormonal therapy with radium-223 (*n* = 167) [51].

In the phase 3b international EAP trial, the efficacy and safety of concomitant treatment with abiraterone/enzalutamide and radium-223 were evaluated in 696 patients from 14 countries; 154 (22 %) received concomitant abiraterone, 50 (7 %) enzalutamide, and 15 (2 %) abiraterone and enzalutamide; 277 (40 %) had prior abiraterone, and 56 (8 %) prior enzalutamide [65]. Baseline characteristics were generally comparable between patients receiving radium-223 alone versus radium-223 with concomitant abiraterone/enzalutamide; median PSA (164.2 vs. 98.9 μg/L) and ALP (161.0 vs. 142 U/L) levels were higher in the former group. Patients receiving radium-223 with concomitant abiraterone/enzalutamide appeared to survive longer than those receiving radium-223 alone. The safety profiles of radium-223 with or without concomitant abiraterone/enzalutamide were similar [65]. A randomized phase 3 trial is under way to confirm these results [65].

With the availability of new agents to improve survival in mCRPC, there is a growing need for data to guide patient selection, choice of combinations, and sequence of administration of these drugs for optimal disease management. Currently ongoing studies with radium-223 are aimed at filling some of these data gaps (Table 2). A randomized, double-blind, placebo-controlled phase 3 trial (ERA223) is currently under way to evaluate the safety and efficacy of radium-223 with abiraterone and prednisone versus abiraterone and prednisone alone in chemotherapy-naïve CRPC patients with metastasis to the bone (Table 2, NCT02043678). It is noteworthy that this trial involves patients who are chemotherapy-naïve and have *asymptomatic* or *mildly* symptomatic mCRPC with bone metastases, a population in which radium-223 has not been used previously. A combination of radium-223 with enzalutamide is currently being tested in asymptomatic or mildly symptomatic CRPC patients with metastases to the bone (Table 2, NCT02194842). Additionally, a combination of radium-223 with the immunotherapeutic sipuleucel-T, which is approved for asymptomatic or minimally symptomatic CRPC with bone metastases, is being investigated in these patients (Table 2, NCT02463799).

#### Conclusions and Future Directions

The beta emitters strontium-89 and samarium-153 did not prolong survival when used as single agents and are recommended solely for pain palliation in mCRPC. The alpha emitter radium-223 improved OS, delayed SSEs, preserved QOL, and was well tolerated in patients with CRPC and symptomatic skeletal metastases. Notably, radium-223 is approved for use in both pre-docetaxel and post-docetaxel clinical settings, with a recommended treatment regimen of 6 cycles. Identification of biomarkers of radium-223 efficacy may optimize disease management, while ongoing clinical studies may clarify treatment sequencing strategies.
